# Neural Plasticity in Spinal and Corticospinal Pathways Induced by Balance Training in Neurologically Intact Adults: A Systematic Review

**DOI:** 10.3389/fnhum.2022.921490

**Published:** 2022-08-17

**Authors:** Yao Sun, Caitlin L. Hurd, Michelle M. Barnes, Jaynie F. Yang

**Affiliations:** ^1^Department of Physical Therapy, Faculty of Rehabilitation Medicine, University of Alberta, Edmonton, AB, Canada; ^2^Neuroscience & Mental Health Institute, University of Alberta, Edmonton, AB, Canada

**Keywords:** postural control, postural equilibrium, exercise, spinal reflexes, corticospinal excitability

## Abstract

Balance training, defined here as training of postural equilibrium, improves postural control and reduces the rate of falls especially in older adults. This systematic review aimed to determine the neuroplasticity induced by such training in younger (18–30 years old) and older adults (≥65 years old). We focused on spinal and corticospinal pathways, as studied with electrophysiology, in people without neurological or other systemic disorders. We were specifically interested in the change in the excitability of these pathways before and after training. Searches were conducted in four databases: MEDLINE, CINAHL, Scopus, and Embase. A total of 1,172 abstracts were screened, and 14 articles were included. Quality of the studies was evaluated with the Downs and Black checklist. Twelve of the studies measured spinal reflexes, with ten measuring the soleus H-reflex. The H-reflex amplitude was consistently reduced in younger adults after balance training, while mixed results were found in older adults, with many showing an increase in the H-reflex after training. The differences in results between studies of younger vs. older adults may be related to the differences in their H-reflexes at baseline, with older adults showing much smaller H-reflexes than younger adults. Five studies measured corticospinal and intracortical excitability using transcranial magnetic stimulation. Younger adults showed reduced corticospinal excitability and enhanced intracortical inhibition after balance training. Two studies on older adults reported mixed results after training. No conclusions could be drawn for corticospinal and intracortical plasticity given the small number of studies. Overall, balance training induced measurable change in spinal excitability, with different changes seen in younger compared to older adults.

## Introduction

Balance training can reduce the incidence of falls in older adults (Sherrington et al., 2008, 2020; Mansfield et al., 2015), and improve postural equilibrium in younger adults (Zech et al., 2010; Lesinski et al., 2015b). Balance training is defined here as training that focuses on improving postural equilibrium in the upright position, while bearing weight on the feet, and performing tasks that challenge the maintenance of the upright posture, i.e., have a tendency to induce falls. Presumably, neural adaptations induced by the training underlie some of the improvements. A few narrative reviews have suggested that both spinal and corticospinal excitability are reduced after balance training in young adults (Taube et al., 2008; Taube, 2012). Another narrative literature review addressing postural control in younger and older adults indicated that there were very few studies addressing neuroplasticity induced by balance training in older adults (Papegaaij et al., 2014). Further, no systematic reviews were found.

Neuroplasticity induced by balance training is not necessarily the same in younger and older adults. The central nervous system changes substantially with age. First, the size of the soleus Hoffmann (H-) reflex and Ia reciprocal inhibition from the dorsiflexors to the soleus is age-dependent (Kido et al., 2004). The H-reflex for the soleus muscle is induced by surface electrical stimulation of the posterior tibial nerve, and measured with surface EMG over the soleus muscle. It measures the excitability in the monosynaptic Ia-motoneuron pathway. The soleus H-reflex is of interest here, because it is highly modulated during various types of movement such as standing, walking and running (Capaday and Stein, 1986, 1987; Edamura et al., 1991), and has been suggested to interfere with upright balance (Llewellyn et al., 1990). The amplitude of the soleus H-reflex is usually normalized to the maximum M-wave (M_MAX_) to control for the natural decline in muscle size with age. Some studies reported lower H_MAX_/M_MAX_ ratio in older adults compared to younger adults (Sabbahi and Sedgwick, 1982; deVeries et al., 1985; Kido et al., 2004; Baudry et al., 2014) while others have reported no changes with age (Vandervoort and Hayes, 1989; Scaglioni et al., 2003). Second, modulation of the H-reflex amplitude as a function of posture also changes with age, even when the levels of background motor neuron excitability is held constant in the tasks. In younger adults, the soleus H-reflex amplitude is largest in lying, and smaller in standing (Koceja et al., 1995; Angulo-Kinzler et al., 1998; Baudry et al., 2015). In contrast, for older adults, the change in the amplitude of the soleus H-reflex as a function of posture was inconsistent. For example, the H_MAX_/M_MAX_ ratio from lying or sitting to standing postures at matched background levels of EMG in the soleus either did not change (Koceja et al., 1995), increased (Angulo-Kinzler et al., 1998) or decreased (Baudry et al., 2015) in older adults.

Motor regions of the brain are also affected by age. Excitability of corticospinal tracts (CST), studied with single-pulse transcranial magnetic stimulation (TMS) to the primary motor cortex, has mostly addressed age-related changes induced in finger muscles (Rossini et al., 1992; Pitcher et al., 2003; Sale and Semmler, 2005; Oliviero et al., 2006; Talelli et al., 2008; Houde et al., 2018; Rozand et al., 2019). In finger muscles, when the amplitude of the motor evoked potential (MEP) is normalized to the M-wave, the maximum MEP amplitude (MEP_MAX_) was either similar between younger and older adults (Rossini et al., 1992; Sale and Semmler, 2005; Rozand et al., 2019), or slightly higher (Pitcher et al., 2003) or lower (Talelli et al., 2008) in the older adults. A small number of studies measured MEP_MAX_ in the leg muscles. Compared to young adults, older adults show greater (Baudry et al., 2014, 2015) or similar MEP_MAX_ (Stevens-Lapsley et al., 2013; Hassanlouei et al., 2017). With a change in posture from sitting to standing, greater MEPs were seen in the soleus muscle at matched levels of background EMG, for both younger and older adults, but the percentages of change from sitting to standing was the same between groups (Baudry et al., 2015). Thus, there may be some differences in how muscles in the upper and lower limbs respond to aging.

Responses to postural perturbations also show age-related differences. For example, the latency of postural responses are slower in older compared to younger adults, with an increase of approximately 5 ms for the short latency response (Nardone et al., 1995), and approximately 20–30 ms for the long latency response (Nardone et al., 1995; Allum et al., 2002; Lin and Woollacott, 2002; Tokuno et al., 2010). The amplitudes of the muscle responses as seen from surface EMG also tend to be larger in older adults, especially in the later responses (Tokuno et al., 2010). Certain types of disturbances, such as lateral platform tilts, reveal interesting differences in strategies between older and younger adults, suggesting that older adults adopt a stiffer trunk posture, perhaps as a result of stiffer passive tissue properties and/or muscle co-contraction, which may predispose them to instability (Allum et al., 2002).

Given these many differences between the young and the old, this systematic review aims to summarize the spinal and corticospinal adaption induced by balance training in people without neurological or musculoskeletal problems. Specifically, we were interested in (1) the neurological changes in the spinal and corticospinal pathways after balance training, and (2) whether older and younger adults show different neural adaption after balance training.

## Methods

The protocol of this systematic review was registered and published on PROSPERO in April 2021 (https://www.crd.york.ac.uk/PROSPERO/display_record.php?RecordID=250604, protocol ID: CRD42021250604).

### Selection Criteria

Original research reported in a peer-reviewed journal were considered. Inclusion and exclusion criteria are outlined in Table 1. We were interested in the plasticity induced naturally by balance training, so training that specifically targeted a pathway was excluded, such as using biofeedback to modify the size of the H-reflex during the training task. The selection criteria for training dosage and frequency were based on previous meta-analyses on the behavioral effects of balance training in younger and older adults (Lesinski et al., 2015a,b). Studies of pathologies were not included to reduce heterogeneity in this initial review. Exceptions were made for studies in which there was mention in the abstract of a control group of neurologically intact individuals. Those papers went through full-text screening to determine if results from the control group were reported separately.

**Table 1:** Inclusion/exclusion criteria.

**Categories**	**Inclusion**	**Exclusion**
Population	Age ≥18 years old with no health concerns. Studies of patient groups that mentioned inclusion of a control group.	Children (<18 years old). Neurological, musculoskeletal or other health-related problems.
Study design	Cohort studies or randomized controlled trials with measures obtained before and after balance training.	Case or case series reports.
Intervention	Balance training that contained: • Challenge to upright balance • Minimal frequency 1x/week • Total number of sessions ≥6	Passive interventions such as vibration, observation or imagining without active participation. Interventions that targeted specific electrophysiological outcomes (i.e., modifying H-reflex amplitude).
Outcome measures in balance	Quantified balance outcomes in standing	Qualitative or self-reported measures of balance
Outcome measures in neuroplasticity	Either one or both of the following: • Spinal excitability as measured by a spinal reflex • Cortical or corticospinal excitability as measured by transcranial magnetic stimulation	Brain imaging measurements only
Language	English language only	Non-English reports

### Search Strategy

Systematic literature searches were conducted in 4 databases: MEDLINE, CINAHL, Scopus, and Embase, under the guidance of a librarian from the University of Alberta. Articles were searched up to April 12^th^, 2021, for MEDLINE, CINAHL, and Scopus, and up to April 14^th^, 2021 for Embase, with no limitation on the start date.

Two sets of keywords were used to encompass the areas of: (1) balance training, and (2) outcome measures of interest. Based on our preliminary search, we found the training programs to be named in a variety of ways, such as sensorimotor training, Tai-Chi training, slackline training, perturbation-based training. Thus, we selected the following keywords to describe the training modality, in an attempt to capture a variety of balance training programs: “balance training” or “balance perturbation” or “functional balance” or “balance exercise” or “Tai-chi” or “agility training” or “alpine skiing” or “sensorimotor training” or “slackline training” or “postural control training” or “unicycle” or “dance” or “martial arts”. Our second set of keywords addressed the outcome measures of interest. We chose to focus on the neural plasticity in spinal and corticospinal pathways, so the keywords were: “neural plasticity” or “training-induced plasticity” or “learning-dependent plasticity” or “brain plasticity” or “neuroplasticity” or “neural adaptation” or “spinal reflexes” or “reflex modulation” or “postural reflex” or “Hoffman reflex” or “cutaneous reflexes” or “musculocutaneous reflexes” or “H-reflex” or “Hmax” or “H-max” or “corticomotor-excitability” or “cortical-excitability” or “spinal-excitability” or “short interval cortical inhibition” or “SICI” or “transcranial magnetic stimulation” or “TMS” or “motor evoked potential”.

Additional filters were used to limit the search results to those studies with human participants, and published in English. The syntax was modified based on the function and interface of each database. Our librarian consultant reviewed and confirmed that the translations of syntax between databases were correct. A complete search syntax is included in the Supplementary Material.

### Screening Process

Search results were exported from the individual databases and imported to Covidence, a management software for systematic reviews (Covidence, Melbourne, Australia). Abstract screening, full-text screening, and data extraction were all completed in Covidence.

The same inclusion/exclusion criteria (Table 1) were used for both abstract and full-text screening. During the abstract screening, two reviewers (YS and one of CH, JY, or MB) screened the titles and abstracts independently to exclude studies that were clearly irrelevant. Studies that met all the criteria, or did not contain sufficient information in the abstract to allow a determination were retained for full-text screening. During the full-text screen, two reviewers (YS and one of CH, JY, or MB) screened the full-text independently. Studies that did not meet the inclusion/exclusion criteria were excluded. Differences between reviewers at each stage of screening were resolved between the two reviewers, and if this was impossible, a third author participated to reach a consensus.

### Data Extraction

Data extraction was conducted by two reviewers (YS and JY, or YS and CH) independently, by reading the full-text. The following data were extracted from the eligible studies: author, title, study purpose, study design, number of participants, sample size justification, age of participants, gender or sex of participants, inclusion & exclusion criteria, the participants' previous experience with the training task, details of the balance training, whether the training was supervised and/or progressive, training frequency (i.e., sessions/week), training dose (i.e., minutes engaged in balance-related tasks), training results (i.e., measures before and after training), measures of spinal pathways, measures of corticospinal pathways, other measures, statistical analyses, conclusions. If the study compared two types of training, such as in a randomized controlled trial (RCT), the group that received the highest dose of balance-related training was used. If both groups received the same amount of balance training, then results from both groups were included.

Further, methodological factors that could influence the electrophysiological outcomes were extracted. For the spinal measurements, these included: (1) nerve and muscle tested, (2) posture during the test, (3) task performed during the test (i.e., at rest or during movement), (4) how the reflex was controlled if the test was performed during movement (i.e., matched background EMG, matched M-wave size or other, or unmatched), (5) stimulus intensity, (6) number of pulses averaged if averaging was used. For TMS measures, these included: (1) the type of coil, (2) coil position, (3) the stimulator used, (4) the number of stimuli averaged, (5) stimulus intensity, (6) conditions that were specifically controlled (such as background EMG, timing of stimulus application), (7) if paired-pulse stimulation was used, the intensity of each pulse and the inter-stimulus interval between the first and second stimulus, (8) the posture during the test, (9) the task performed during the test.

### Quality Assessment

The Downs & Black checklist (Downs and Black, 1998) was used to assess the quality of each study. This method encompasses evaluation of methodological quality for both RCT and non-RCT studies. The score reflects the quality of the reporting, internal and external validity. Two evaluators (either YS and CH or YS and JY) independently scored the papers and discrepancies were resolved by discussion and consensus.

### Synthesis of Evidence

Studies were grouped according to the ages of the participants, based on the mean age. Those studies reporting a mean age of 65 years old or older were considered older adults. The intervention methods differed considerably, so the interventions were grouped according to similarities in type, for example, balancing in standing on unstable surfaces, martial arts, and specific types of sports. Since most of the training sessions included some time spent in warm-up, rest, or exercises that did not focus on balance, we calculated the total time spent in balance-related exercises as follows. The aspects of the training that challenged balance were identified, and the time spent on those balance tasks were calculated. This required that the study provided description of the tasks and the time spent on each of the tasks.

The balance outcomes also differed considerably, so we determined whether a quantitative outcome demonstrated improvement, such as displacement of the center of pressure during standing on a force platform, or a standardized and validated clinical score. Outcomes of spinal and corticospinal excitability were grouped according to the type of measure and variable quantified. In cases where a comparable outcome was reported from multiple groups, the data were summarized in table or graphic form. If means and standard deviations were not reported or only reported in graphic form, then the corresponding authors were contacted to obtain the actual means and standard deviations. If authors did not respond after two attempts to contact, the means were estimated directly from the reported figures.

## Results

### Study Selection and Characteristics

**Figure 1:**
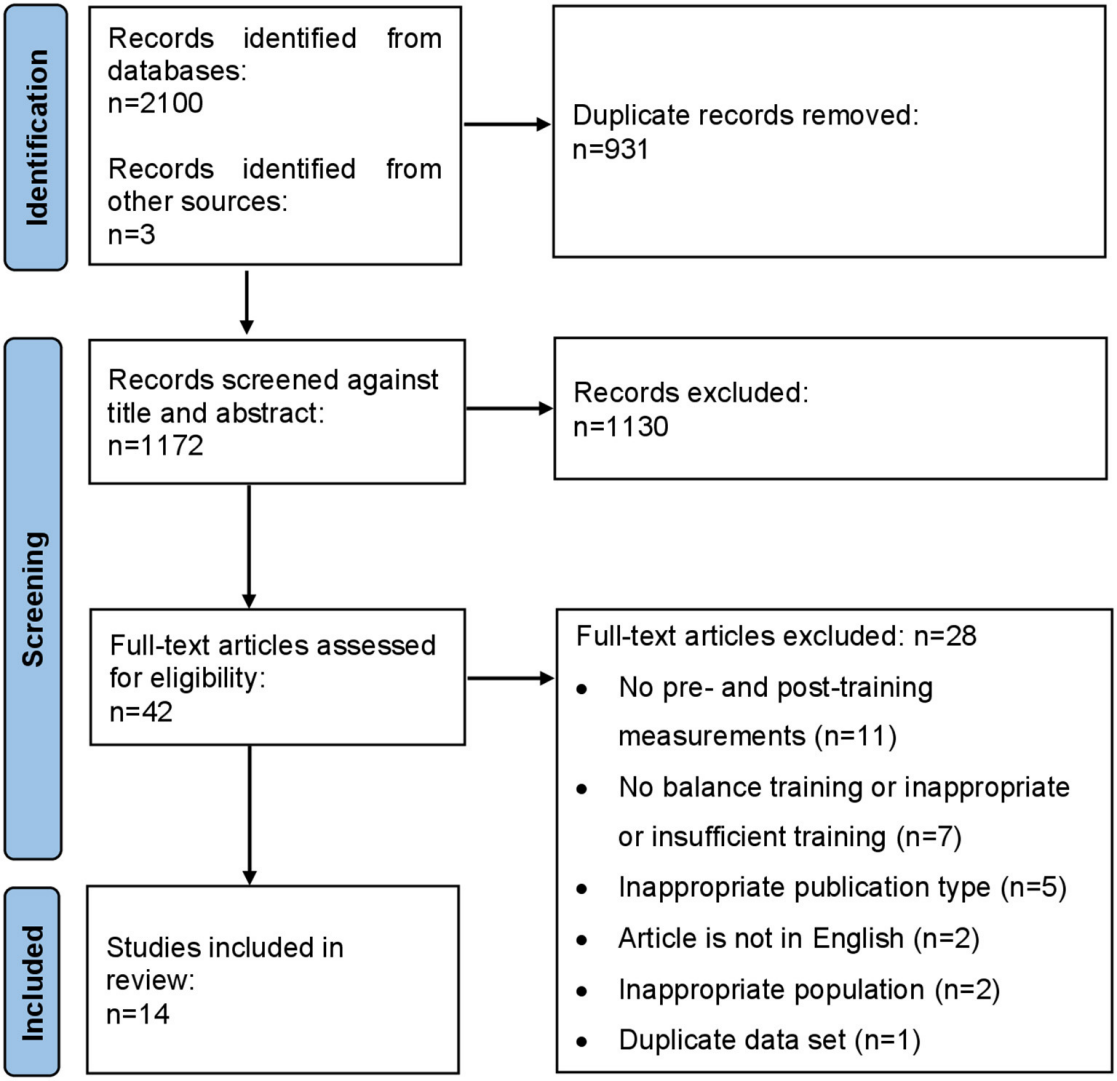
Flowchart of systematic search and article inclusion and exclusion process.

Figure 1 shows the PRISMA flow diagram. A total of 2,103 articles were identified. After removing 931 duplicates, we screened 1,172 abstracts and 42 articles were included for full-text screening. The majority of papers were excluded because they were irrelevant, or did not meet our inclusion criteria. During full-text screening, one paper was identified (Granacher et al., 2006) to have measured balance using methods that were not independent of the electrophysiological measures. In this study, postural reflexes were induced by translating the support-surface (treadmill), and improvements in balance (reduction in induced ankle and knee velocity) as well as neuroplasticity (reflex latencies) were deduced from these same trials. We excluded this paper, because we believe the argument was circular, in that any changes in neurophysiological outcome would likely induce changes in the mechanical outcome of the same trial, and could not be assumed to be an improvement in balance without an independent measure of balance, as reported in all other papers included in our review. We did not anticipate this possibility a priori, but by consensus between the authors, this paper was excluded. Thus, full-text screening resulted in 14 eligible studies.

Among the 14 studies (Table 2), 12 measured spinal plasticity, 5 measured corticospinal or intracortical plasticity, including 3 that measured both spinal and corticospinal plasticity. Key data extracted from measures of spinal excitability and corticospinal or intracortical plasticity are shown in Tables 3, 4, respectively. One paper (Lauber et al., 2021) presented results from both soleus H-reflex and TMS measurements, but the data collection procedures for the H-reflex were not reported. Thus, this study was included among those that only measured corticospinal plasticity; the results of H-reflexes from this study were not included. Six out of 14 studies trained older adults (65 years or older); these studies are identified in blue shading in Tables 2–4. One study did not specify the ages of the participants (Keller et al., 2012), but we confirmed the age of the participants to be younger adults through communication with the corresponding author. Thus 8 studies focused on younger adults between 18 and 30 years old.

**Table 2:** Summary of participant characteristics, training, balance outcomes, and the quality of the papers.

**Type**	**Study**	**Participants & group chosen**	**Training methods & training time**	**Balance measurements & results**	**D&B**
Standing balance: unstable surfaces	Gruber et al., 2007	*n =* 11, 4 females, 26 ± 5yr Experience: none Group: sensorimotor training	**Methods**: Standing on 4 devices - wobbling board, spinning top, soft mat, and cushion. Uni-pedal standing (right). Eyes opened. **Training dose**: 1 hr/session, 4 sessions/week, 4 weeks for a total of 16 sessions. Sessions include warm-up, cool down and rest time. Balancing for 20s X 4 trials/device, progressed to 6 trials/device. **Balancing time:** Sessions #1-8: 5.3 min/session (20sx4x4); Sessions #9-16: 8 min/session (20sx4x6), 107 min in total.	**Balance measurements:** Displacement of a moveable platform during uni-pedal standing (right). Participants stood with the right knee bent to about 30°, hands akimbo, and view directed to a nearby wall. 40s/trial X 3 trials. Cumulative displacement was calculated. **Results:** ↓displacement of the platform	13
	Taube et al., 2007	*n =* 13, 5 females, 25 ± 3yr. Experience: none Group: sensorimotor training	Same as Gruber et al. (2007) (above)	**Balance measurements:** Displacement of a moveable platform during uni-pedal standing (right). 40s/trial X 3 trials. Cumulative displacement and the average of 3 trials were calculated. The peak-to-peak amplitude of vertical ground reaction force (GRF_V_) over 500 ms window after backward movement of the treadmill during quiet stance. **Results:** ↓ displacement of the platform and GRF_V_	14
	Behrens et al., 2015	*n =* 13, 6 females, 24.6 ± 2.5yr. Experience: none Group: intervention	**Methods**: Standing on 7 different balance training devices. Start with bipedal standing progressing to uni-pedal standing and/or closed eyes, then performing additional motor tasks during balancing. **Training dose:** 1 hr/session, 2 sessions/week, 8 weeks for a total of 16 sessions. Balancing for 30s/trial, progressing to 40s/trial then 60s/trial X 4 trials/device. **Balancing time:** 14 min/session (30sX4X7) for 2 wk, 18.6min/session (40sX4X7) for 2 wk, and 28min/session (60sX4X7) for 4 wk, 354 min in total.	**Balance measurements:** Postural sway measured by a force plate. Bipedal standing on 1) rigid surface and 2) foam surface with eyes open and fixating on target. 30s/trials, 3 trials/condition. The mean of COP_AP_, COP_ML_ from each trial and the mean of 3 trials was calculated. **Results:** One of COP_AP_ or COP_ML_ reduced, but not specified which.	16
	Penzer et al., 2015	*n =* 8, 6 females, 71.4 ± 6.4 yr. Experience: not mentioned Group: greater balance training	**Methods:** Standing on 3 devices - a rigid surface, foam, and BOSU (half circle inflated ball) balance trainer, with 2 different visual conditions (eyes open, eyes closed), and 5 different feet positions (normal, joined, tandem, single leg on each leg), i.e., 30 different exercises in total. Difficulty was increased by changing the feet position first, then performing the same exercises with eyes closed. Thereafter, the same progression for feet and vision conditions was applied when balancing on the foam and on the BOSU. Progression was only made when participants can perform 3 trials of the same exercise without losing balance. **Training dose**: 1hr/session, 2 sessions/week, 6 weeks for a total of 12 sessions. Sessions included warm-up and cool down. One session/wk of balance entirely: 30s X 3trials per exercise, ~10 exercises, PLUS 1 session/wk of strength and balance, in which 6 balance exercises were practiced 1 trial each, 30s/trial. **Balancing time:** 15min/session (30sX3X10) for 1 session/wk, 3 min/session (30sx1x6) for 1 session/wk, 108 min in total.	**Balance measurements:** Postural sway measured by a force plate. Bipedal standing on 1) rigid surface, eyes open, 2) rigid surface, eyes closed, 3) foam surface, eyes open. COP was calculated with 10s moving windows throughout 40 s of standing. Maximum and standard deviation of COP excursion in anteroposterior and mediolateral directions (COP_AP_, COP_ML_, COP_AP−SD_, COP_ML−SD_) were calculated. Averaged rectified EMG in TA, SOL, GAS. **Results:** ↓ COP_AP_ and COP_AP−SD_ during standing on foam. ↓ EMG in plantarflexors in standing conditions (which plantarflexor muscle was not specified).	14
	Ruffieux et al., 2017	*n =* 15, 8 females, 70.1 ± 4.4yr. Experience: not mentioned Group: balance training	**Methods:** Standing on 4 devices - foam pad, tilt board, air-filled cushion, and spinning top balance board. Uni-pedal standing. Both legs were trained. **Training dose:** Duration of each session was not reported. Three sessions/week, 5 weeks for a total of 15 sessions. Sessions include balancing for 20s X 4 trials/device/leg. **Balancing time:** 10.7min/session (20sX4X4X2), 160min in total	**Balance measurements**: Postural sway measured by a force plate during uni-pedal (right) and bi-pedal stance on a solid surface. The number of errors during uni-pedal standing and total COP pathway (COP_TOTAL_) was calculated. Displacement of a moveable platform during bipedal standing. In 15s/trial, 3 trials/condition, the best of 3 was used for statistical analysis. **Results:** ↓error during uni-pedal stance. ↔ COP_TOTAL_, displacement of the platform.	14
	Mouthon and Taube, 2019	*n =* 13, 4 females, 24 ± 3yr (age of the training vs control groups not separated). Experience: not mentioned Group: balance training	**Methods:** Standing on an unstable platform that tilted laterally. Bipedal standing. **Training dose:** 55min/session, 2 sessions/week, 3 weeks for a total of 6 sessions. Sessions include 10 mins warm-up. Balancing 30s X 15trials/ platform. **Balancing time:** 7.5 min/session (30sX15), 45min in total	**Balance measurements:** Time (s) that the participant could keep the freely moving platform within ±5 deg of the horizontal. 3 trials in total. **Results:** ↑ balancing time during balancing test	13
	Lauber et al., 2021	*n =* 24 (10 females) 23.3 ± 2.4yr. Experience: not mentioned Group: both groups	**Methods:** Standing unipedal on 4 devices - two-dimensional swinging platform, wobble board, spin top, soft mat. Both legs trained in turn. Difficulty was increased by adding repetitions, closing the eyes, and catching a ball while balancing. One group received balance training followed by strength training, the other group received strength training then balance training. **Training dose**: Duration of each session was not reported. 3 sessions/week, 4 weeks, for a total of 12 sessions. Balancing 30s/trial, progressing from 3 to 6 trials/device. **Balancing time:** 12 min/session (30sX3X4X2) to 24 min/session (30sX6X4X2). Unclear when the progression in time occurred. Total balancing time: 144-288 mins.	**Balance measurements:** Displacement of a moveable platform. Uni-pedal (right) standing: 1) for 30s as still as possible, 2) to counterbalance anteroposterior perturbations from the platform for 20s. 3 trials/condition, cumulative sway of the platform was calculated and averaged. **Results:** ↓ displacement of the platform (the study did not specify which task this result is from)	17
Standing balance: stable surface with self-initiated movement	Maejima et al., 2009^a^	*n =* 26, 13 females, 69.8 ± 0.5yr. Experience: not mentioned Group: exercise group	**Methods:** Standing balance exercise with hands holding a chair. Training includes 55mins exercise session plus at least 30mins of walking. 55mins exercise includes 15mins of abdominal breathing exercises, 20mins active stretching, 10mins leg exercise in standing, 10mins leg exercise in sitting, 10mins plantarflexion exercises in sitting. **Training dose:** Daily exercise for 3 months, ~ 90 sessions in total. Only standing leg exercise is considered to be balance training. **Balancing time:** 10min/session; 754 min in total. Total balancing time was calculated based on the average attendance rate reported in the article. Group training: 94.5% X 6days X 10 min = 56.7min Home exercise: 83.0% X (90-6) days X 10min = 697.2min	**Balance measurements:** Postural sway measured by a force plate. Dynamic balance: bipedal standing with sudden forward and backward translations of a force platform, 5 trials/direction. Static balance ^1^: bipedal standing with 1) eyes open, 2) eyes closed (30s/trial, 1 trial/condition); Voluntarily movable lengths of COP in forward, backward, left and right directions. Berg Balance scale items: 1) one-leg standing time, and 2) functional reach; **Results:** ↔ maximum COP excursion and time to maximum COP excursion after perturbation, one-leg standing time. ↑ COP excursion in static standing with eyes open/closed, functional reach distance, voluntary movable length of COP in the left and right directions.	15
	Freyler et al., 2014	*n* = 32, 18 females, 24 ± 2 yr. Experience: not mentioned Group: both groups	**Methods:** Standing on left leg keeping COP (visual feedback provided) as still as possible. Standing on both legs and controlling the COP to trace a circle on the screen as accurately as possible. 16 participants received regular training, 16 participants trained with 60% of body weight unloaded **Training dose:** 35mins/session, 2 sessions/week, 4 weeks for a total of 8 sessions. Sessions include 10mins static balance training and 20min dynamic balance training. Each task was performed for 30s with pauses of 30s. **Balancing time:** ~15min/session, 120min in total	**Balance measurements:** Postural sway measured by a force plate during 1) bipedal standing; 2) uni-pedal stance on a stable surface; 3) uni-pedal standing on an unstable surface. COP_AP_, COP_ML_, and COP_TOTAL_, were calculated. Participants stood with hands akimbo looking forward. 30s/condition, 3 trials/condition, the average of 3 trials was calculated. **Results:** ↓COP_TOTAL_ during one-leg stance on stable and unstable surfaces	13
Martial Arts	Chen et al., 2011	*n =* 20, 9 females, 72.9 ± 4.4yr. Experience: none in Tai Chi Group: training	**Methods:** Tai Chi training was delivered by a qualified Tai Chi instructor. The “13-form” Yang style Tai Chi was practiced at a slow pace. **Training dose** 1 hr/session, 3 sessions/week, 12 weeks for a total of 36 sessions. Sessions include 20 min warm-up and 40 min Tai-Chi practice. **Balancing time:** 40 min/session; 1,440 min in total	**Balance measurements:** Postural sway measured by a force plate, with 4 bipedal standing conditions on (1) stable surface with eyes open, (2) stable surface with eyes closed, (3) unstable surface with eyes open, and (4) unstable surface with eyes closed. 10s/trial, 10 trials/condition. COP_AP_, COP_ML_, and the average of the 10 trials were calculated. **Results:** ↔ COP_AP_, COP_ML_ in all conditions	20
	Ma et al., 2019	*n =* 17, 2 females, 67.5 ± 6.3 yr. In 13 participants completed training Experience: not regularly engaged in martial arts or other training. Group: Ving Tsun group	**Methods:** Ving-Tsun training was delivered by a certified coach. Nine sets of drills were practiced with a partner, 20 repetitions/ drill. **Training dose**: 1 hr/session, 2 sessions/week, 12 weeks for a total of 24 sessions. Sessions include 40-50min of Ving Tsun training. **Balancing time:** 40-50min/session, ~960-1080min in total	**Balance measurements:** COP displacement and velocity in a 5s window after manually induced perturbation (assessor pushes participant from the back ~T1-T2 level). Activities-Specific Balance Confidence score, the number of falls in the 3 months of training. **Results:** ↔ COP displacement length or velocity, Activities-Specific Balance Confidence score, the number of falls in the three months of training.	23
Slackline	Keller et al., 2012	*n =* 12, 6 females, 23.3 ± 1.0yr. Experience: none (unable to balance on slackline >20s). Group: training	**Methods:** Slackline training. Difficulty was increased by reducing assistance while walking on the slackline, changing the length and the tension of the slackline, and performing a motor task while balancing on the slackline. **Training dose**: 1.5hr/session, 2–3 sessions/week, 4 weeks for a total of 10 sessions. After every 2 min on the slackline, participants rest for 2 min. **Balancing time:** ~45min/session on the slackline; 450 min in total.	**Balance measurements:** Slackline performance: 3 trials of balancing standing on the slackline for 20 s, measured as successful or not successful. Standardized displacement of a moveable platform during bipedal standing on the platform (number and duration of trials were not mentioned). Displacement of the platform over 15s measured. **Results:** Improved slackline performance (none could balance for at least 20s without assistance before training, and all could balance after training). ↓ Displacement of the platform in the mediolateral direction after perturbations.	10
	Giboin et al., 2019	*n =* 22, 10 females, 25 ± 4yr. Experience: none Group: Training	**Methods:** Slackline training. Difficulty was increased by reducing support, walking on the slackline, changing the length of the slackline, performing a motor task while balancing on the slackline, etc. **Training dose**: 45 min/session, 2 sessions/week, 6 weeks for a total of 12 sessions. Sessions include 5 mins warm-up and 40mins of training **Balancing time:** 40min/session; 480 min in total	**Balance measurements:** Number of steps taken on the slackline: participants had to stand on one leg for at least 2 s before starting the next step. Time of balancing on a tilt-board with right leg. **Results:** ↑ steps on the slackline. ↔ time on tilt-board.	14
Alpine ski	Lauber et al., 2011^b^	*n =* 22 Reflex test: *n =* 13, 6 females, 66.8 ± 2 yr. Balance test: *n =* 21, 8 females, 67.1 ± 2yr. Experience: intermediate skiers Group: intervention	**Methods:** Guided skiing in a group of 'homogeneous level' skiers, with one guide. **Training dose**^2^: Actual skiing time reported to be 67.6 ± 7.2 min/session, 2–3 sessions/week, 12 weeks for a total of 28.5 ± 6 days of skiing on average. **Balancing time:** 67.6 min/session, 1927 min in total or ~32 h.	**Balance measurements:** Postural sway measured by a force plate. Bipedal stance, eyes open for 30s. **Training outcomes:** ↓ Postural sway	17

*Studies were organized according to broad categories of training types (Column 1 – Type). Those focused on older adults are shown with blue shading. Participant characteristics include their prior experience with the training method (Experience). If the studies had more than one group of participants, the group chosen (Group) for this review is indicated (see text for how groups were chosen). The balance results refer to the changes from before to after training. Quality of the paper was evaluated with the Downs & Black (D&B) checklist (Downs and Black, 1998). N, number of participants; yr, years old, means ± SD; ms, milliseconds; s, seconds; min, minutes; hr, hours; ↑, increased; ↓, decreased; ↔, no change; COP_TOTAL_, total excursion of the center of pressure; COP_AP_, center of pressure excursion in the anteroposterior direction; COP_ML_, center of pressure excursion in the mediolateral direction; T1-T2, vertebral thoracic level 1 and 2; **Results** refers to the change in outcomes after training compared to before training.*

a *Static balance measurements of Maejima et al. (2009) were obtained from an article published by the same group on the same project (Maejima et al., 2008)*.

b *Training dose of Lauber et al. (2011) was obtained from an article published by the same group on the same project (Müller et al., 2011)*.

**Table 3:** Spinal excitability - measurements and results.

**Type**	**Study**	**Spinal measurements**	**Results**
Standing balance: unstable surfaces	Gruber et al., 2007	**SOL H-reflex and stretch reflex** **Task:** sitting at rest **Test methods:** H-reflex and M-wave recruitment curves and H_MAX_/M_MAX_ were obtained. Stretch reflex was induced by passive dorsiflexion with an ergometer, stretch amplitude was 6° at a velocity of 200°/s. Mean peak-to-peak values from 40 stimuli were calculated.	↓H_MAX_/M_MAX_ ratio, stretch reflex amplitude/ M_MAX_ ratio ↔ M_MAX_ ↔ Stretch reflex latency
	Taube et al., 2007	**SOL H-reflex** **Task:** bipedal standing on a stationary treadmill with perturbation applied by posterior translation of the treadmill belt. **Test methods**: tibial nerve stimulation was timed to occur at the short (SLR) and long-latency (LLR) postural responses after the perturbation. Latency of SLR and LLR were determined in separate trials. H-reflex and M-wave recruitment curves and H_MAX_/ M_MAX_ ratio were obtained at each latency.	↔ SLR, LLR, H-reflex latency. ↓ H_MAX_/M_MAX_ ratio No correlation found between balance outcomes and changes in H_MAX_/M_MAX_ ratio
	Behrens et al., 2015	**SOL H-reflex** **Task:** sitting 1) at rest, 2) 2 s after initiation of a maximum isometric contraction (MVC) of the SOL. **Test methods:** H-reflex and M-wave recruitment curves and H_MAX_/M_MAX_ ratio were obtained at rest. Then, H-reflex was evoked during the MVC with the stimulation intensity required for H_MAX_ when at rest. M-wave and V-wave were evoked during the MVC with stimulation intensity 40 % greater than that needed for a maximal twitch response and concomitant M_MAX_.	↓ H_MAX_/M_MAX_ at rest ↔normalized V-wave and normalized H-reflex evoked during MVC.
	Penzer et al., 2015	**SOL H-reflex** **Task:** bipedal standing on a stable surface. **Test methods:** H-reflex and M-wave recruitment curves and H_MAX_/M_MAX_ ratio were obtained. The H-reflex recruitment curve was fit to a sigmoid curve, and stimulus intensity at 50% H_MAX_ (H_50_), 50% M_MAX_ (M_50_), H_MAX_, M_MAX_, and slope of the recruitment curve at H_50_ (H_SL_) were calculated.	↔ M_MAX_, H_MAX_/M_MAX_ ratio, stimulus intensity at H_MAX_ ↓ H_SL_, H_50_ Results of stimulus intensity at 50% M_MAX_, and M_MAX_ were not reported.
	Ruffieux et al., 2017	**SOL H-reflex** **Task:** bipedal standing on a stable surface. **Test methods:** H-reflex and M-wave recruitment curves and H_MAX_/M_MAX_ ratio were obtained. Background EMG was calculated as root mean square from a 100 ms window before each stimulus.	↔ H_MAX_/M_MAX_ ratio ↔ Background EMG
Standing balance: stable surface with self-initiated movement	Maejima et al., 2009	**Postural reflexes and integrated EMG after perturbation** **Task:** bipedal standing on a force plate **Test methods:** Perturbations were elicited by forward and backward translations of a force platform. Translations were at 100 mm/s, 50 mm distance. EMG latency was calculated from the onset of perturbation to activation of each muscle. Integrated EMG was calculated from a 1s window from the activation of the muscle and normalized to the EMG during isometric MVC of the same muscle. EMG of TA, QUAD, GAS, and HAM was measured.Five trials per direction were averaged.	Forward platform displacement: ↓EMG of TA, QUAD, HAM ↓ latency of HAM & GAS ↔ latency of TA & QUAD, EMG of GAS. Backward platform displacement: ↓EMG of TA & QUAD ↓ latency of GAS ↔ latency of TA & QUAD, EMG of GAS & HAM.
	Freyler et al., 2014	**Soleus H-reflex** **Task:** 1) bipedal standing on a stable surface, uni-pedal standing on a 2) stable surface, 3) unstable surface. **Test methods:** H-reflex and M-wave recruitment curves were obtained during standing. Then stimulation intensity was controlled to generate an M-wave at 25% M_MAX_, and 20 stimuli were applied for each condition. H-reflex from uni-pedal standing tasks were normalized to the H-reflex obtained during the bipedal stance condition.	↓ normalized H-reflex in one-leg standing tasks ↔ M_MAX_
Martial arts	Chen et al., 2011	**Soleus H-reflex** recruitment curves during quiet standing. **Task:** bipedal standing on (1) stable surface with eyes open, (2) stable surface with eyes closed, (3) unstable surface with eyes open, and (4) unstable surface with eyes closed. **Test methods:** H-reflex and M-wave recruitment curves and H_MAX_/M_MAX_ were obtained from each condition. H_MAX_ averaged over 10 trials for each condition.	↑ H_MAX_/M_MAX_ ratio in all four conditions
	Ma et al., 2019	**Postural reflex after perturbation** **Task:** bipedal standing with blindfolded **Test methods:** Perturbation was manually induced (assessor pushes participant from the back ~T1-T2 level). Postural reflex latency was determined as the onset of trunk acceleration to the onset of muscle activity in HAM and GAS of the dominant leg. Only one trial was performed.	↑ EMG onset time of GAS ↔ EMG onset time of HAM
Slackline	Keller et al., 2012	**SOL H-reflex** **Task:** 1) bipedal standing on a stable surface, and uni-pedal standing on 2) Posturomed, 3) air cushion, 4) slackline. **Test methods:** H-reflex and M-wave recruitment curves and H_MAX_/M_MAX_ ratio were obtained from each condition. Background EMG was calculated as root mean square in a 1.5–3.5s window after each stimulus during the recording of the recruitment curve test then normalized to M_MAX_.	↓ H_MAX_/M_MAX_ ratio in all conditions ↔ non-normalized or normalized background EMG.
	Giboin et al., 2019	**Soleus H-reflex** **Task:** Uni-pedal standing (right) on slackline and tilt-board. **Test methods**: stimulation was applied within a window of 100 ms before to 20 ms after foot touchdown on slackline/tilt-board. Stimulation intensity was controlled to generate an M-wave that was 10% M_MAX_. H-reflex amplitude was normalized to M_MAX_. Background EMG was calculated as the root mean square of a 50 ms window before the stimulation.	↓ normalized H-reflex amplitude during slackline task ↔ background EMG
Alpine ski	Lauber et al., 2011	**SOL H-reflex and paired reflex depression** **Task:** 1) prone, 2) bipedal standing on a stable surface, 3) bipedal standing on an unstable surface. Participants' position during PRD test was not mentioned. **Test methods:** H-reflex and M-wave recruitment curves and H_MAX_ /M_MAX_ ratio were obtained from each condition. For paired reflex depression, inter-stimulus intervals were 10s and 1s (10 pulses/condition), stimulus intensity was controlled to generate an M-wave that was 20% M_MAX_, the 2nd to 10th reflexes were averaged. Background EMG of TA, SOL, GAS was calculated as the root mean square from 1.5–3.5 s after each stimulus	↑ H_MAX_/M_MAX_ ratio during unstable standing ↔ paired reflex depression (no numbers or graphs presented) ↔ background EMG

*Changes in spinal reflexes from before to after training. Studies focused on older adults are shown with blue shading. Studies are grouped according to training type, as in Table 2. ms, milliseconds; s, seconds; EMG, electromyography; SOL, soleus; TA, tibialis anterior; GAS, gastrocnemius; QUAD, quadriceps; HAM, hamstrings; H_MAX_, maximum amplitude of the H-reflex; M_MAX_, maximum amplitude of the M-wave. The following symbols refer to changes seen after compared to before training in all the variables listed after the symbols: ↑ - increased; ↓ - decreased; ↔ - no change*.

**Table 4:** Corticospinal and intracortical excitability - measurements and results.

**Type**	**Study**	**Corticospinal measurements**	**Results**
Standing balance: unstable surfaces	Taube et al., 2007	**SOL MEP and TMS conditioned H-reflex** **Task:** bipedal standing on a stationary treadmill, with perturbation applied by a posterior translation of the treadmill belt (15 cm displacement, 60 m/s^2^ acceleration). **Test methods**: 1) Single-pulse TMS evoked MEPs at 0.9 aMT, timed to occur during the SLR and LLR after the perturbation. 2) TMS conditioned SOL H-reflex: stimuli were timed to arrive at the SLR and LLR during perturbations. At least 16 response/latency were recorded and averaged.	↓MEPs and TMS conditioned H-reflex at LLR. ↔ MEPs and TMS conditioned H-reflex at SLR.
	Ruffieux et al., 2017	**SOL TMS conditioned H-reflex** **Task**: same as Taube 2007 (above) **Test methods:** same as Taube 2007 (above) except the stimuli were timed to arrive at LLR only, and stimulation intensity was at rMT.	↔ TMS conditioned H-reflex at LLR.
	Penzer et al., 2015	**SOL MEP**_**MAX**_**, and MEP recruitment curve**. **Task:** bipedal standing. **Test methods**: single-pulse TMS with stimulation intensity increased in steps of 4-10% maximal output of the stimulator (MSO), 5 stimuli per intensity until MEP_MAX_ or 100% MSO reached. Stimulus intensity at 50% MEP_MAX_ (MEP_50_) and slope at MEP_50_ (MEP_SL_) were calculated.	↔ MEP_MAX_, stimulation intensity at MEP_50_ ↓ MEP_SL_ ↑ Stimulus intensity to evoke MEP_MAX_.
	Mouthon and Taube, 2019	**TA SICI** **Tasks:** Performed in the following order: bipedal standing on 1) stable platform, 2) tilting platform with elastic straps constrain the tilt, 3) freely tilting platform, and 4) lying. **Test methods**: conditioning stimulation at 0.8 aMT, test stimulation at 1.2 aMT, with aMT measured during Tasks 2 & 3, above. Inter-stimulus interval: 2.5 ms. For each standing task, 20 unconditioned and 20 conditioned stimuli were averaged. For the lying condition, rMT was first determined, then conditioning (0.8 rMT) and test (1.2 rMT) stimuli were delivered as above, with 48 unconditioned and 48 conditioned stimuli averaged.	↑ SICI in all standing conditions ↔ SICI when measured in lying; ↑ aMT ↔ MEP from unconditioned stimulus
	Lauber et al., 2021	**SOL MEP, SICI**, **Task:** 1) ballistic plantarflexion in sitting, 2) bipedal standing on a stationary treadmill, and perturbation applied by posterior translation of the treadmill belt (displacement 21 cm, acceleration 1.6 m/s^2^, 3) rest sitting, 4) bipedal standing. **Test methods**: SICI: conditioning stimulation at 0.8 MT, test stimulation at 1.2 MT, with aMT measured for Task 1 &2 and rMT measured for Task 3 & 4. Interstimulus interval: 2.5 ms. The background EMG for ballistic plantarflexion and balancing perturbation were matched when TMS was triggered. 15 unconditioned and 15 conditioned stimuli were averaged. MEP evoked from unconditioned stimulus during Task 1&2 were normalized to M_MAX_ and compared before and after training	Strength training followed by balance training: ↔ SICI after balance training Balance training followed by strength training: ↑SICI during the balancing task after balance training. Both groups: ↔ aMT, rMT, normalized MEP during Task 1&2.

*Changes in corticospinal measurements from before to after training. Studies focused on older adults are shown with blue shading. Studies are grouped according to training type, as in Table 2. ms, milliseconds; s, seconds; ↑, increased; ↓, decreased; ↔, no change; SOL, soleus; TA, tibialis anterior; GAS, gastrocnemius; TMS, transcranial magnetic stimulation; MEP, motor evoked potential; MEP_MAX_, maximum MEP; SICI, Short interval intracortical inhibition; rMT, resting motor threshold; aMT, active motor threshold; SLR, short latency response; LLR, long latency response; EMG, electromyography*.

### Quality of the Studies

The scores obtained from the Downs & Black checklist are shown in Table 2 (last column). Higher scores indicate better quality, with the best possible score being 28. The majority of papers were of moderate quality: mean ± standard deviation (SD) = 15.2 ± 3.3, median = 14, with a range from 10 to 23. The most common unmet criterion was blinding of participants to the intervention, which was impossible by nature of the interventions. Other criteria that were most often unmet were: reporting of adverse events, using a representative sample of the population, delivering the intervention in a setting where such interventions are normally delivered, blinding of evaluators, and concealment of randomization to participants and researcher until completion of the study.

### Training Modality and Dosage

The training details are summarized in Table 2. Studies were broadly grouped into 5 categories: (1) standing balance on unstable surfaces (7 studies), (2) standing balance on a stable surface (2 studies), (3) martial arts training (2 studies), (4) slackline training (2 studies), and (5) alpine skiing (1 study). Here, the term “standing balance” describes training that required participants to stand on one spot while maintaining balance during various tasks. All the training exercises were performed in an upright posture, supporting body weight on the feet.

Total training time ranged from 6 sessions over 3 weeks to daily exercise over 3 months. All studies provided sufficient information to estimate the actual time spent on balance-related tasks except the skiing time of Lauber et al. (2011), which was obtained from a different article using the same training method, referred to by the authors in their Methods (Müller et al., 2011). In this way, the total balancing time through the entire training program was estimated to range from 45 min (Mouthon and Taube, 2019) to 32 h (Lauber et al., 2011). The frequency of exercises ranged from 2 to 7 times per week, and the duration of the sessions ranged from about 5 min to 3 h per session.

### Balance Outcomes

Balance measurements and results are summarized in Table 2. Most of the studies provided sufficient detail about balance outcomes. The study by Maejima and colleagues (Maejima et al., 2009) referred to an earlier publication for methods regarding the balance measure, so they were obtained from the earlier publication (Maejima et al., 2008). Balance outcomes were measured in a variety of ways. For example, balance duration when standing uni- or bipedally on different surfaces, postural sway when attempting to stay stationary, the degree of disturbance to the body from a perturbation, or the reach distance in a Functional Reach Test (Duncan et al., 1990). Regardless, the majority of studies showed improvements in balance with training. Only two of the 14 studies did not show improvements (Chen et al., 2011; Ma et al., 2019).

### Spinal Plasticity After Balance Training

Spinal plasticity was reported in 12 studies with two types of spinal reflexes: (1) soleus H-reflex (10 studies), and (2) short latency postural reflexes induced by external perturbations (2 studies). Measurement methods and results from spinal reflexes are shown in Table 3.

Soleus H-reflexes were quantified in three different ways: (1) the ratio of maximal H-reflex over the maximum M-wave (H_MAX_/M_MAX_) (8 studies); (2) the H-reflex during a task normalized to another standardized testing condition or M_MAX_ (normalized H-reflex) (2 studies); (3) the slope of the rising phase of the H-reflex recruitment (1 study). Since there were several papers that reported H_MAX_/M_MAX_ under matched conditions before and after training, we summarized these pre-post changes for younger and older adults. In cases where more than one condition was reported for the H_MAX_/M_MAX_, H_MAX_/M_MAX_ ratios from all the conditions were extracted. All authors contacted to obtain values responded except two, so those were estimated directly from the published figures.

**Figure 2:**
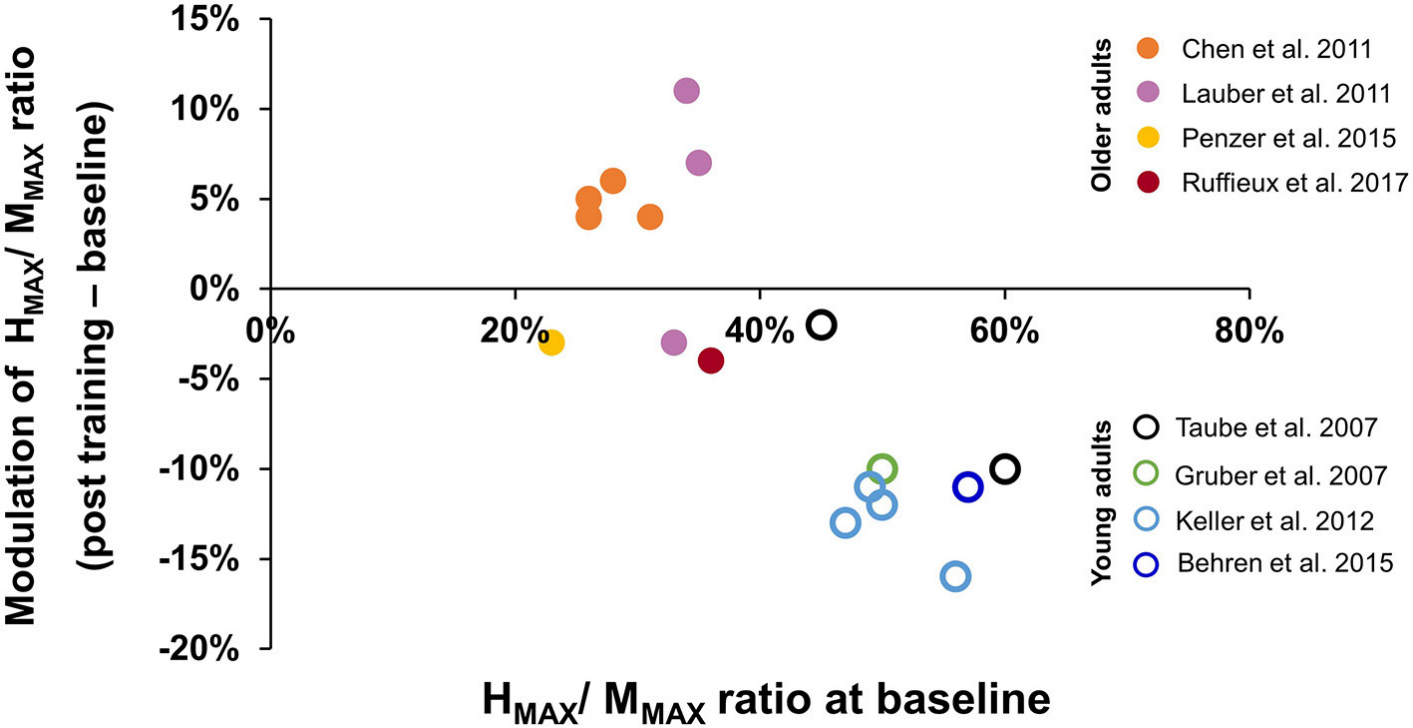
Modulation of H_MAX_/M_MAX_ ratio after balance training. The x-axis represents averaged H_MAX_/M_MAX_ ratio at baseline, the y-axis represents the modulation of H_MAX_/M_MAX_ ratio after balance training, with negative values indicating reduced H_MAX_/M_MAX_ ratio after training. Data were obtained from 8 studies (see Results, Section 3.5). Data from different test conditions within the same study are represented by the same color. The solid dots represent data from older adults, while the open circles represent data from young adults.

The results for the change in H_MAX_/M_MAX_ are shown in Figure 2, with cold colors (dots) representing younger adults and warm colors (open circles) representing older adults. Note that some studies reported the findings under more than one condition, so those different conditions are represented with the same color. As a group, older adults had smaller H-reflexes prior to training compared to younger adults (horizontal axis); this was expected based on prior literature (see Introduction). Interestingly, the way in which the reflexes changed with balance training was also different. Younger adults tended to show a reduction in the H_MAX_/M_MAX_ with training, whereas older adults tended to show smaller changes in either direction, with a suggestion that the change depended on the magnitude of H_MAX_/M_MAX_ prior to training.

Postural reflexes after perturbations were measured in two studies with older adults. The perturbations were applied through translational movements of a platform in anterior or posterior directions or through a forward push from the experimenter. One study reported shorter response latency in the gastrocnemius muscle, and decreased integrated EMG from the TA and quadriceps muscles (Maejima et al., 2009) while the other study showed greater reflex latency in the gastrocnemius muscle (Ma et al., 2019).

### Corticospinal and Intracortical Plasticity After Balance Training

Corticospinal or intracortical excitability was reported in five studies, with four of the five studies coauthored by a few of the same authors, and originating from two labs. Methods and results are summarized in Table 4.

One study reported the input-output relationship of the MEP amplitude in the soleus muscle during standing in older adults only (Penzer et al., 2015). The slope of the input-output relationship was reduced and the stimulus intensity required to elicit the MEP_MAX_ increased after training, suggesting a reduction in the excitability of the motor cortex (Penzer et al., 2015).

Two studies measured TMS-conditioned soleus H-reflex in response to standing perturbations (Taube et al., 2007; Ruffieux et al., 2017). Perturbations were applied through a standardized, rapid backward movement of the treadmill belt at 60 m/s^2^, while the participant stood on the stationary treadmill belt. In these two studies, TMS conditioned H-reflexes were used to indicate the excitability of the monosynaptic input from the CST. The soleus H-reflex and the single-pulse, subthreshold, TMS-evoked MEP in the soleus were timed to arrive during the short (SLR) or long-latency response (LLR) after the perturbation. The inter-stimulus interval between the stimulation to the tibial nerve and primary motor cortex was timed so that the TMS pulse arrived at the motor neuron when the first observable H-reflex facilitation occurred. Facilitation at this time is mostly attributable to the direct monosynaptic projections from the motor cortex to spinal motor neuron (Nielsen et al., 1993; Petersen et al., 1999). The amplitude of the TMS conditioned H-reflex reduced after balance training at the LLR but not the SLR in younger participants (Taube et al., 2007). Further, a significant correlation was observed between improvements in balance performance (i.e., postural sway during uni-pedal standing) and reduction in TMS-conditioned H-reflex after perturbations. The results suggest a reduction in the corticospinal input to the soleus with training in younger participants, and that the reduction was associated with the improvements in balance. With the identical training and assessment methods, the above changes were not observed in older participants, even though there were improvements in balance, as reflected by a uni-pedal stance test (Ruffieux et al., 2017).

Two other studies (Mouthon and Taube, 2019; Lauber et al., 2021) reported changes in short latency intracortical inhibition (SICI) after training in younger participants. SICI is thought to reflect GABA_A_ activity in the motor cortex (Ziemann, 2013). SICI was measured in the TA (Mouthon and Taube, 2019) and SOL muscles (Lauber et al., 2021), using standard protocols (Kujirai et al., 1993) that were identical in the two studies. Briefly, after the hotspot for obtaining MEPs in each muscle was determined, paired-pulse TMS was used, with the initial conditioning pulse subthreshold (0.8x active motor threshold), and the subsequent test pulse suprathreshold (1.2x active motor threshold), and an inter-stimulus interval of 2.5 ms in both studies. SICI was quantified as the amount of depression generated by the conditioning pulse as a function of the size of the unconditioned test pulse. In one study (Mouthon and Taube, 2019), SICI in the TA muscle was elicited under 3 conditions: lying, quiet standing, and standing on different movable platforms. Enhanced SICI was observed in all the test conditions after balance training. SICI in the SOL muscle was elicited under 4 conditions: quiet sitting, sitting with ballistic plantarflexion, quiet standing, and standing with perturbation from posteriorly moved treadmill belt. Increased SICI was only observed in the soleus muscle after balance training during the standing with perturbation condition (Lauber et al., 2021). Overall, greater intracortical inhibition was observed when a change was induced after training.

## Discussion

### Overview

In general, balance training induced different excitability changes in the spinal reflexes in younger compared to older adults. The amplitude of the soleus H-reflex, for example, was consistently reduced in younger participants after balance training, whereas the same reflex tended to change less or in the opposite direction after training in older adults. A number of possible reasons for these differences are considered below. Corticospinal adaptations were reported in five studies with only two studies focused on older adults. Because of the small number of studies, and very few in which younger and older adults were tested under matched conditions, no clear conclusions can be drawn with respect to cortical and corticospinal excitability.

### Possible Explanations for Differences in Training Induced Changes in Spinal Reflexes Between Younger and Older Adults

Balance training in younger adults resulted in a reduction of the soleus H-reflex gain after training, most clearly seen in the change to the H_MAX_/M_MAX_ ratio (Figure 2). A reduction in the soleus H-reflex gain with balance training is consistent with acute modulation of the reflex gain when balance is challenged. For example, comparing lying to standing (Mynark and Koceja, 1997; Mynark et al., 1997), sitting to standing (Goulart et al., 2000), or standing to walking (Capaday and Stein, 1986), decreased soleus H-reflex amplitude was observed when balance was challenged, at matched excitability levels of the motor neuron pool (i.e., matched background EMG). Studies that incorporated balancing on unstable surfaces induced further reductions in the H-reflex amplitude (Llewellyn et al., 1990; Hoffman and Koceja, 1995; Solopova et al., 2003). Some have suggested that reduced spinal reflex gain can prevent the automatic soleus stretch reflex from precipitating postural instability, thereby enhancing balance during a more challenging task (Llewellyn et al., 1990). Perhaps the reduction in reflex gain with training serves the same purpose.

Compared to younger adults, older adults showed smaller H-reflex amplitudes before training (Figure 2), again consistent with other reports of the soleus H-reflex in older adults, regardless of posture (Sabbahi and Sedgwick, 1982; Koceja et al., 1995; Angulo-Kinzler et al., 1998; Baudry et al., 2015), even when normalized to the maximum M-wave (Koceja et al., 1995; Angulo-Kinzler et al., 1998; Baudry et al., 2015). Interestingly, the changes induced by balance training in older adults were either a very small decrease, or an increase in the H_MAX_/M_MAX_ ratio, opposite in direction to that seen in younger adults (Figure 2). The reasons for these differences between younger and older adults are unknown.

One explanation might be that modulating the reflex gain with training may be more difficult to achieve in older adults, analogous to “blunted” neuromuscular plasticity induced by resistance training (Bickel et al., 2011). There is limited evidence, however, that older adults have the same capacity as younger adults to reduce the gain of the soleus H-reflex when it is appropriate for balance. For example, when the H-reflex was used as a perturbation during standing on an unstable surface, older adults were able to reduce their H-reflex amplitude to the same extent as younger adults, at least in the short term (i.e., over 2 training days) (Mynark and Koceja, 2002).

Another possibility is that there is a particular range of H-reflex gain that is best suited for a particular task, and hence the direction and magnitude of the change are dependent on the baseline excitability prior to training, which could vary between individuals. This idea has been proposed by Koceja et al. (1995), when comparing the H-reflex modulation from prone to standing. In their study, young participants showed a reduction in H_MAX_/ M_MAX_ from prone to standing. In contrast, older adults exhibited two types of responses: the subgroup with higher H_MAX_/ M_MAX_ in prone showed a reduction in H_MAX_/ M_MAX_ when standing, while the subgroup with lower H_MAX_/ M_MAX_ in prone showed an increase in H_MAX_/ M_MAX_ when standing (Koceja et al., 1995), such that both groups reached similar H_MAX_/ M_MAX_ in the standing position. With long term training in a balancing task, as we are considering here, it is possible that the magnitude and direction of change in the soleus H-reflex is dependent on the baseline H-reflex excitability prior to training. There is considerable individual variation between participants in the excitability of the spinal circuits, as shown by Solopova et al. (2022). Further, although aging is associated with a general reduction in the H-reflex amplitude (reviewed in Introduction), it may also be modified by the level and type of activity the individual normally engages in Pérot et al. (1991), Raglin et al. (1996). This may partially explain why balance training led to a different magnitude and direction of change among older adults.

### Cortical Motor Pathways After Balance Training

After balance training in younger participants, there was a reduction in the excitability of the corticospinal pathways to the soleus muscle (Taube et al., 2007), and an increase in the SICI to the tibialis anterior (Mouthon and Taube, 2019) and soleus (Lauber et al., 2021). Together, this suggests that with training, the role of the motor cortex may become smaller in balance tasks in younger adults, especially during tasks that are similar to the trained tasks. The results are less clear in older adults, since the slope of the input-output curve for MEP was reduced during stationary standing (Penzer et al., 2015), and the TMS conditioned H-reflex was unchanged during balancing tasks (Ruffieux et al., 2017).

One caveat is that the methods used to obtain some of these findings were extremely complex. For example, the paradigm to measure TMS-conditioned H-reflexes during the LLR induced by a perturbation required that the perturbation, the occurrence of the LLR, the peripheral nerve stimulation and the TMS pulse had to be perfectly timed (Taube et al., 2007; Ruffieux et al., 2017). Further, the background excitability of the spinal motor neurons at the time of occurrence of these stimuli would have to be the same before and after training to truly reflect a change at the cortical level. Their measure of the excitability of the motor neuron pool was based on the background EMG obtained in separate trials with the mechanical perturbation alone without the stimulation, and from the background EMG 200 ms before the onset of the perturbation. These measures do not necessarily guarantee that the excitability of the motor neuron pool was the same for either the separate trials, or for measures taken at different times after the onset of the perturbation.

Brain activity as seen from functional MRI during imagined movements of the lower extremity showed increased brain activation in older compared to younger adults, especially during imagined standing (Zwergal et al., 2012) and isolated ankle movements (Sacheli et al., 2020). This suggests that the brain regions involved during lower extremity movements change with age. Further, neuroplasticity in the motor regions of the brain may decline with age, as seen from an age-dependent reduction in the transcranial theta-burst TMS induced long-term depression (LTD) in the primary motor cortex (Freitas et al., 2011); see also a broader review of age-related changes in brain plasticity in Pascual-Leone et al. (2011). Thus, it is very likely that there are differences between younger and older adults in how they respond to balance training and how well the learned behaviors are retained. These gaps in our knowledge remain to be explored.

### Limitations

This review included only English publications, and four databases were searched. Thus, it is possible that relevant papers were missed. Further, some papers reporting balance training in individuals with pathology may have contained control participants, but were screened out because the inclusion of control participants was not mentioned in the abstract. This review was focused on electrophysiological measures of plasticity, so brain plasticity measured with brain imaging was not included.

The training methods were diverse among the included papers. We attempted to reduce the variability by focusing on training methods that were carried out during weight-bearing on the feet with a purpose to challenge balance, and had a minimum dosage (6 sessions) and frequency (once per week). Nevertheless, they ranged from exercises during standing, martial arts, skiing, and slackline walking. With this broad range of exercises included, only 14 studies fit our inclusion/exclusion criteria. Thus, it was impossible to focus more narrowly on specific training methods. It is likely that the different forms of exercise induced different neuroplastic changes, many of which may not have been possible to extract from these small numbers of diverse papers. Further, there was no measure of training intensity, a gap that remains to be filled by future work.

The diversity of outcome measures used both for balance and neurophysiological measures were considerable, making it difficult to conduct meta-analyses. We confirm that the conditions of measurement, such as the task performed during the electrophysiological measurements, the stimulus intensity used, among others, were matched in each study before and after training. Nevertheless, it is likely that the measurement conditions contributed to some of the variability in the findings. Finally, the moderate quality and the small number of papers mean that the conclusions remain preliminary.

## Data Availability Statement

The original contributions presented in the study are included in the article/Supplementary Material, further inquiries can be directed to the corresponding author.

## Author Contributions

YS and JY conceived of the study and designed the search approach. YS searched articles. YS, CH, MB, and JY screened abstracts and full texts. YS, CH, and JY extracted data. The draft of the manuscript was written by YS and JY, and revised by YS, CH, MB, and JY. All authors read and approved the final manuscript.

## Funding

YS is funded by a Postdoctoral Recruitment Award from the Faculty of Rehabilitation Medicine at the University of Alberta.

## Conflict of Interest

The authors declare that the research was conducted in the absence of any commercial or financial relationships that could be construed as a potential conflict of interest.

## Publisher's Note

All claims expressed in this article are solely those of the authors and do not necessarily represent those of their affiliated organizations, or those of the publisher, the editors and the reviewers. Any product that may be evaluated in this article, or claim that may be made by its manufacturer, is not guaranteed or endorsed by the publisher.
