# Supplementary material for: Neural Plasticity in Spinal and Corticospinal Pathways Induced by Balance Training in Neurologically Intact Adults: A Systematic Review
Source: Front Hum Neurosci. 2022 Aug 17;16:921490. doi: 10.3389/fnhum.2022.921490 (PMC9428930; doi:10.3389/fnhum.2022.921490)
Supplement: Supplementary file 1 [file Data_Sheet_1.DOCX]

**Appendix**

**Search syntax for MEDLINE**

1 ((balance adj4 training) or balance perturbation or functional balance or balance exercise*).mp.

2 (Balance and (exercise* or Tai-chi or agility training or alpine skiing or rehabilitation or physiotherapy or physical therapy or (sensorimotor adj4 training) or slackline training or postural control or (postural adj4 training) or unicycl* or danc* or martial arts)).mp.

3 1 or 2

4 cortical excitability/ or evoked potentials, motor/ or evoked potentials, somatosensory/ or neuronal plasticity/ or h-reflex/ or reflex, stretch/

5 Transcranial Magnetic Stimulation/

6 (((Neural or training-induced or learning-dependent or brain) adj4 plasticity) or neuroplasticity or neural adaptation or ((spinal or modulation or postural or Hoffman or cutaneous or musculocutaneous) adj3 (reflex or reflexes)) or H-reflex* or Hmax* or H-max* or corticomotor excitability or spinal excitability or SICI or short interval cortical inhibition or transcranial magnetic stimulation or TMS or motor evoked potential).mp.

7 4 or 5 or 6

8 3 and 7 526

9 limit 8 to animals

10 limit 9 to humans

11 8 not (9 not 10)

12 limit 11 to english language

**Search syntax for CINAHL**

( ( ( ((balance N4 training) or balance perturbation or functional balance or balance exercise*) ) OR ( (Balance and (exercise* or Tai-chi or agility training or alpine skiing or rehabilitation or physiotherapy or physical therapy or (sensorimotor N4 training) or slackline training or postural control or (postural N4 training) or unicycl* or danc* or martial art)) ) ) ) AND ( ( (MH "Transcranial Magnetic Stimulation") OR ( (MH "Reflex, Stretch") OR (MH "H-Reflex") OR (MH "Neuronal Plasticity") ) OR ( (MH "Evoked Potentials, Motor") OR (MH "Evoked Potentials, Somatosensory") ) OR ( (((Neural or training-induced or learning-dependent or brain) N4 plasticity) or neuroplasticity or neural adaptation or ((spinal or modulation or postural or Hoffman or cutaneous or musculocutaneous) N3 (reflex or reflexes)) or H-reflex* or Hmax* or H-max* or corticomotor-excitability or cortical-excitability or spinal-excitability or SICI or short interval cortical inhibition or transcranial magnetic stimulation or TMS or motor evoked potential)) ) )

**Search syntax for Scopus**

( TITLE-ABS-KEY ( ( ( balance W/4 training ) OR balance-perturbation OR functional-balance OR balance-exercise* OR ( balance AND ( exercise* OR tai-chi OR agility-training OR alpine-skiing OR rehabilitation OR physiotherapy OR physical-therapy OR ( sensorimotor W/4 training ) OR slackline-training OR postural-control OR ( postural W/4 training ) OR unicycl* OR danc* OR martial-art* ) ) ) ) AND TITLE-ABS-KEY ( ( transcranial-magnetic-stimulation OR ( ( neural OR training-induced OR learning-dependent OR brain ) W/4 plasticity ) OR neuroplasticity OR "neural adaptation" OR ( ( spinal OR modulation OR postural OR hoffman OR cutaneous OR musculocutaneous ) W/3 ( reflex OR reflexes ) ) OR h-reflex* OR hmax* OR h-max* OR corticomotor-excitability OR cortical-excitability OR spinal-excitability OR sici OR short-interval-cortical-inhibition OR tms OR motor-evoked-potential ) ) ) AND NOT ( TITLE ( rat OR rats OR pig OR pigs OR porcine OR mouse OR mice OR hamster OR hamsters OR {animal} OR {animals} OR bovine OR sheep OR murine OR primate* OR zebra* OR drosphil* ) OR ( KEY ( rat OR rats OR pig OR pigs OR porcine OR mouse OR mice OR hamster OR hamsters OR {animal} OR {animals} OR bovine OR sheep OR murine OR primate* OR zebra* OR drosphil* ) AND NOT KEY ( human* ) ) )

**Search syntax for Embase**

1 ((balance adj4 training) or balance perturbation or functional balance or balance exercise*).mp.

2 (Balance and (exercise* or Tai-chi or agility training or alpine skiing or (sensorimotor adj4 training) or slackline training or postural control or (postural adj4 training) or unicycl* or danc* or martial arts)).mp.

3 1 or 2

4 cortical excitability/ or evoked potentials, motor/ or evoked potentials, somatosensory/ or neuronal plasticity/ or h-reflex/ or reflex, stretch/

5 Transcranial Magnetic Stimulation/

6 (((Neural or training-induced or learning-dependent or brain) adj4 plasticity) or neuroplasticity or neural adaptation or ((spinal or modulation or postural or Hoffman or cutaneous or musculocutaneous) adj3 (reflex or reflexes)) or H-reflex* or Hmax* or H-max* or corticomotor excitability or spinal excitability or SICI or short interval cortical inhibition or transcranial magnetic stimulation or TMS or motor evoked potential).mp.

7 4 or 5 or 6

8 3 and 7 492

9 limit 8 to animals

10 limit 9 to humans

11 8 not (9 not 10)

12 limit 11 to english language
